# Editorial: Entrepreneurial psychological capital and spirituality: A core distinction among entrepreneurs

**DOI:** 10.3389/fpsyg.2023.1125826

**Published:** 2023-02-01

**Authors:** Clara Margaça, Jose Carlos Sánchez-García, Lisete M. Mónico, Helena Knörr

**Affiliations:** ^1^Department of Social Psychology and Anthropology, University of Salamanca, Salamanca, Spain; ^2^Faculty of Psychology and Educational Sciences, University of Coimbra, Coimbra, Portugal; ^3^Rowland School of Business and the Department of Literary Arts and Social Justice, Point Park University, Pittsburgh, PA, United States

**Keywords:** psychological capital, entrepreneurship, university students, psychological resources, pandemic (COVID-19)

## Introduction

Humanity is going through a troubled period at health, social, and economic levels. Entrepreneurs are actors suited to operate in an increasingly competitive world, as they are able to face challenges in a distinctive way and turn them into opportunities. What are the differentiating characteristics of these people?

Positive Psychology consistently asserts that positivity drives people to become forward-looking entrepreneurs to achieve a meaningful life and make communities more prosperous. Combining Positive Psychology with educational curricula allows potential entrepreneurs to improve and know how to apply positivity, creativity, empathy, and critical thinking to entrepreneurial issues and bringing changes to society (Luthans et al., 2013). In this sense, several studies suggest that psychological capital endows entrepreneurs with greater self-confidence, hope, optimism, and greater flexibility, which allows them to achieve personal and professional fulfillment. Psychological capital is a psychological state composed of four dimensions: optimism, resilience, hope, and efficacy (Luthans et al., 2007, 2013). According to Luthans et al. (2007), it is extremely important for entrepreneurs to be able to maintain a positive mindset, because it allows them to successfully face obstacles (resilience). According to the precursor of psychological capital, hope is another value with a decisive character for entrepreneurs. An entrepreneur with hope is able to persevere in the goals and, if necessary, act and make the necessary effort (self-efficacy) to redirect them. Several authors (e.g., Hizam-Hanafiah et al., 2017) even consider psychological capital to be a very important resource for an entrepreneur, an extension of the concept of “economic capital” (Luthans and Youssef, 2004). In addition, it is a capacity that originates from the cognitive functions that determine attitudes and that entrepreneurs can develop (Tang, 2020), allowing for increased chances of success in business activity (Luthans et al., 2007). Therefore, positive psychology increases individual and, consequently, organizational performance.

Currently, the study of entrepreneurship has revealed a new and greater understanding of the motivations, personal values, and belief structures that influence a person's decision to become an entrepreneur (Bird and Schjoedt, 2009; Krueger, 2009). We can mention a psychology of entrepreneurship, which focuses on understanding the relationship between successful business leadership and the cognitive and individual characteristics of successful entrepreneurs.

From another perspective, we are pleased to mention the spiritual mindset and its importance in the business environment, starting with decision-making (Neubert et al., 2017). The implementation of spiritual values in the organizational environment is seen as an evolutionary process that connects the entrepreneur with a higher level of meaning. Zsolnai (2022) argues that the development of a spiritually informed economics can support the flourishing of life, through the implementation of ecological and humane economic practices. According to Rehan et al. (2019), spirituality sharpens the entrepreneur's sense of community. The entrepreneur who is guided by his spiritual, moral and social values will find, innately, but also creatively and flexibly, solutions for the improvement of society (Kurt et al., 2020; Agarwal et al., 2022).

This Research Topic aimed to provide a discussion of new research ideas and present trends development on positive psychology and spirituality on entrepreneurship, and totals 8,792 views and 1,283 downloads. In addition, it totals 24 authors, from five different countries. The collected articles (*N* = 7) in this Research Topic are summarized in Table 1.

**Table 1:** Summary of contributions.

**No**.	**Authors**	**Title**	**Purpose**	**Views**	**Cit**.
1	Sheng and Chen	The effect of COVID-19 College Students' Entrepreneurial Intentions	Based on the premise that the COVID-19 pandemic has brought a large number of entrepreneurial opportunities, the objective of this study was to analyze whether this fact attracts university students to the path of entrepreneurship. The authors supported their study on the following three theories: event system theory, regulatory focus theory, and cognitive appraisal of emotion theory.	2,538	1
2	Barrientos Oradini et al.(a)	Curiosity as a moderator of the relationship between entrepreneurial orientation and perceived probability	In this study, the authors sought to examine whether the two different types of curiosity moderate the relationship between entrepreneurial orientation and the likelihood of starting a business, considering their interaction with sociodemographic variables.	1,181	3
3	Jiatong et al.	From COVID-19 Pandemic to entrepreneurial behavior: the mediating effect of proactive personality and the moderating role of anticipated regret	The aim of this article is essentially to assess whether the COVID-19 pandemic had an impact on entrepreneurial intention. In addition, the variables proactive personality and anticipated regret were used as a mediator and moderator, respectively.	1,615	1
4	Lin et al.	The impact of entrepreneurial spirituality on business performance: based on the survey of private enterprise executives in Fujian China	In a mass entrepreneurial and innovation context, in China, in this article, the authors sought to analyze whether entrepreneurial spirituality influences the performance of top executives.	720	2
5	Crespí et al.	The challenge of developing entrepreneurial competence in the University using the Project-oriented Learning Methodology	With a sample of university students from Madrid, the authors studied the development of entrepreneurial skills through the implementation of the project-oriented learning methodology.	605	1
6	Zhang et al.	An empirical analysis of double reduction education policy based on public psychology	In this study, the authors analyzed the impact that the double reduction policy can have on the way people think and process information about the education system.	1,659	1
7	Barrientos Oradini et al.(b)	Passion and perseverance: how the components of grit affect the probability of starting a business	In this research work, the authors assessed the differences that the variables age, gender and culture can assume in the decision-making process of becoming an entrepreneur. In addition, the concepts of Grit-Perseverance and Grit-Passion were introduced to study their relationship with the variables Entrepreneurial Orientation and Probability of Opening a Business.	489	1

In the first article, Sheng and Chen used a mediation model combining the following theories: event system theory, regulatory focus theory, and cognitive appraisal theory of emotion. The results showed that the effect of the pandemic period on entrepreneurial intention is mediated by all variables [i.e., (1) defensive regulatory focus and fear of failure; and (2) facilitated regulatory focus and fear of failure] on an ongoing basis. Furthermore, the findings indicate that the factors that influence entrepreneurial intentions can be broader and, for this reason, future research should incorporate the role of education for entrepreneurship, as well as entrepreneurship policies.

Next, Barrientos Oradini et al.(a) distinguish two types of curiosity, both at a theoretical and empirical level: the first—i-type—concerns an epistemic curiosity, the desire to obtain new knowledge in order to stimulate intellectual interest; the second—D-type—refers to the elimination of information deprivation conditions (Litman, 2012). The authors examined the moderation effect that I-type and D-type curiosity have on the relationship between Entrepreneurial Orientation and Probability of Starting a Business, considering their interaction with several sociodemographic variables. These authors present curiosity as a psychological factor. The results show that when D-type curiosity presents higher values, the association between Entrepreneurial Orientation and Probability of Starting a Business is stronger. In terms of sociodemographic variables, only age had a moderated effect of D-type curiosity on the relationship between Entrepreneurial Orientation and Probability of Starting a Business. Moreover, the authors discussed the results highlighting the spiritual attitudes, practices and behaviors and problem-solving inherent in any entrepreneurial activity. Furthermore, they conclude that D-type curiosity has elements common to psychological capital, in particular with regard to self-efficacy and resilience. These elements are decisive and influential to start a new business.

The aim of the study conducted by Jiatong et al. was to analyze the impact of COVID-19 on the student's entrepreneurial intention using the proactive personality as a mediator and the anticipated regret as a moderator. The authors concluded that the perception of COVID-19 negatively influences students' entrepreneurial intention. The pandemic crisis caused by COVID-19 highlighted the existing problems in the labor market that young people face and demonstrated how vulnerable this group is in times of crisis. Furthermore, the findings show that a proactive personality is an important measurer between COVID-19 perception and entrepreneurial intention. It was also possible to verify that anticipated regret positively and significantly moderates the relationship between intention and entrepreneurial behavior. A proactive personality can be decisive for achieving happiness, because when the environmental situation is uncertain, in a pandemic crisis, people with a high level of proactive personality are more likely to resort to their innovative skills and see difficulties as an opportunity to undertake.

Based on the Chinese entrepreneurial reality, Lin et al. analyzed whether entrepreneurial spirituality influences executive performance. The authors developed the Entrepreneurial Spirituality and Business Performance questionnaire, which allowed them to determine that responsibility has the highest score, followed by innovation, proactivity and risk-taking. Furthermore, it is women who score higher on Entrepreneurial Spirituality. In addition, it was possible to highlight that: Entrepreneurial Spirituality has a positive impact on business performance; different factors of Entrepreneurial Spirituality have different effects on business performance, and innovation and proactivity play the biggest role. Finally, it is possible to concluded that there is an inverted U-shaped relationship between innovation, risk taking and business performance.

Crespí et al. study analyzed whether the development of skills such as achievement orientation, proactivity, planning, team work, and management can be influenced by the project-oriented learning methodology. A single sample of university students from Madrid and a quasi-experimental pre-test/post-test methodology was used. The different analyzes carried out showed that students have a positive and significant self-perception of their entrepreneurial skills and competences.

Zhang et al. sought to analyze how people process information in relation to the Chinese education system, through SEM and under the implementation of the double reduction policy. The results highlight the importance of a fair public educational system, above all, through the performance of training institutions.

Finally, Barrientos Oradini et al.(b) seek to examine whether grit-passion and grit-perseverance play a moderating role in the relationship between Entrepreneurial Orientation and the Probability of Starting a Business, and concluded that only the first type of grit has this role. In addition, it was also found that none of the sociodemographic variables showed a moderating effect. The authors conclude their study by drawing a parallel between Grit-passion and the four dimensions of psychological capital: effectiveness, hope, optimism and resilience. People with this characteristic are more oriented toward achieving goals, are able to see opportunities in difficulties and are able to overcome barriers.

## Contributions and future directions

The articles in this Research Topic were positioned across three main academic fields: economics and management, psychology and education, and embracing a broad range of topics related to psychological capital and entrepreneurship. Entrepreneurship is the main vector of economic development, and offers the possibility of social advancement to different segments of the population. The economic crisis of recent years has led to an exponential rise in unemployment, especially among the youngest groups in society (Švarcová and Horáková, 2015). If the environment of uncertainty can be a barrier to economic activity, the entrepreneur has proven to be a key element in leveraging economic and social development. According to the literature, hope, effectiveness, resilience, and optimism can be seen as a psychological resource and determinant for starting a business activity (Carr, 2011). Furthermore, in an organizational environment where psychological capital is implemented, it is possible to achieve better levels of wellbeing, commitment and productivity at work. Overall, the articles gathered in this Research Topic have highlighted the importance of psychological factors, above all, as predictors of entrepreneurial intention.

## Author contributions

CM drafted the first version of the editorial. JS-G, LM, and HK made several contributions to the reformulation of the first draft. All authors provided conceptual input and approved the final draft.
